# Physical Inactivity in Older Adult Couples: A Dyadic Analysis Using Continuous In-Home Monitoring

**DOI:** 10.1093/geroni/igaa057.1327

**Published:** 2020-12-16

**Authors:** Chao-Yi Wu, Lyndsey Miller, Rachel Wall, Zachary Beattie, Jeffrey Kaye, Lisa Silbert

**Affiliations:** 1 Oregon Health & Science University, Portland, Oregon, United States; 2 Oregon Health and Science University, Portland, Oregon, United States; 3 Portland Veterans Affairs Medical Center, Portland, Oregon, United States; 4 Layton Alzheimer’s Disease Center, Portland, Oregon, United States

## Abstract

Many older adults remain inactive despite the known positive health implications of physical activity (e.g. improved mood, reduced mortality risk). Physical inactivity is a known interdependent phenomenon in couples, but the majority of research identifies determinants of physical inactivity at the individual level. We estimated the average amount of physical inactivity for older adult couples and, using dyadic analysis, identified physical and mental health determinants thereof. Forty-eight heterosexual older adult couples (mean age=70.6, SD=6.63) from the Veterans Integrated Service Network 20 cohort of the Collaborative Aging Research using Technology (CART) initiative were included in this study. Both dyad members wore actigraph devices for a month. The average number per day of inactive periods (defined as no movement or sleep activity for ≥ 30 minutes) was estimated. Multilevel modeling revealed that, within couples, there was no difference between partners in the average number of inactive periods, but on average across couples, males had more inactive periods per day (13.4, SD=4.43) than females (12.3, SD=4.87). For males, older age was the only variable associated with more inactive periods (β=0.13, p=.013). For females, more depressive symptoms in men were associated with fewer inactive periods (β=-0.37, p=.002), and more dependence in completing their own IADLs predicted more inactive periods (β=2.80, p<.001). All models were adjusted for covariates. Viewing couples’ activity as a unit, rather than as separate individuals, provides a novel approach to identifying pathways to reduce inactivity in older adults, especially when focusing on mental health issues and decreased independence within the couple.

